# A Study on Embodied Experience of Surfing Tourism Based on Grounded Theory—Take China’s Hainan Province as an Example

**DOI:** 10.3390/bs12110407

**Published:** 2022-10-22

**Authors:** Shenyang Zhang, Yangle Chen

**Affiliations:** School of Tourism, Hainan University, Haikou 570228, China

**Keywords:** surfing tourism, embodied experience, grounded theory

## Abstract

In the context of the rapid development of surfing tourism in China, the behavior explanation of surfing tourists has not attracted attention from the academic circle. Based on the theory of embodiment, this study takes surfing tourism in Hainan Province as the first case to explain the process and results of body experience in surfing tourism behavior. Based on the grounded theory analysis of the collected online travel notes and on-site interview text materials related to tourism experience, 21 categories and 6 main categories were extracted, and the story line of the surfing tourism experience was constructed based on embodied experience. The results show that the embodied phenomena and processes of the surfing tourism experience affect the quality of tourists’ experience. Surfing tourists experience four typical processes, namely embodied perception, embodied awakening, embodied emotion and embodied extension, and represent the body’s meaning, self-identity and social value through surfing behavior. The research theoretically proposes the embodied experience model and a new category of surfing tourism and provides a reference value for the practice of the surfing tourism industry.

## 1. Introduction

Surfing is a traditional behavior with a long history, and modern surfing is an extreme sport involving riding the waves with a surfboard [1]. At present, the number of tourists seeking surfing experiences is increasing rapidly around the world. In China, surfing tourism is rapidly developing as a new Marine tourism product, which has a profound impact on the Marine tourism experience of Chinese tourists. Since surfing was included in the Olympic events in 2016, Riyue Bay in Wanning, Hainan, China, has become the training base for the surfing national team. Compared with Western countries, surfing in China has a late start, low popularity and low public awareness, but some tourists have tried surfing out of curiosity and adventure and shared their experiences on social media, which has won the attention of a large number of potential tourists. As a result, the number of surfing tourists in Wanning City, Hainan Province, has increased year by year, and it is known as China’s surfing “resort”. At present, the total number of members of Riyue Bay Surfing Club has grown from more than 1000 in 2011 to nearly 20,000, which fully reflects the attraction of surfing tourism in Wanning [2].

However, the rapid development of surf tourism in China has not received the academic attention it deserves, with only little academic research in China focusing on technical aspects such as concept introduction, market development and regional planning. Empirical research on the theoretical aspects of tourists’ surfing behavior has been neglected. Surfing tourism needs to master certain skills and has certain dangers, allowing people the opportunity to challenge themselves and immerse themselves in nature. At the same time, surfing tourism requires a high level of physical fitness and balance in order to master the fast-changing waves and to complete the surfing experience. For surfers, the behavior process of adjusting themselves and conquering nature is also the behavior of continuously constructing the meaning of the tourism experience. The development of embodied theory has brought a new paradigm shift for tourism experience research [3]. Different from the visual gaze of the tourism experience, the embodied theory emphasizes the integration of the presence of the body and the “field”, to obtain a holistic tourism experience.

Therefore, in this study, the body experience of tourists motivated by surfing behavior needs to be described and explained theoretically. By taking this as the logical starting point, this study describes the embodied experience phenomenon of surfing tourists, explains its embodied experience mechanism and interprets the significance of the surfing tourism experience through coding and analyzing the text of the surfing tourism experience in on-site interviews and online tourism sharing.

## 2. Literature Review

### 2.1. The Formation and Development of Surfing Tourism

The study on surfing tourism is rooted in the practice of surfing sports and the development of surf tourism destinations. The case studies mainly focus on well-known surfing resorts such as Australia, Hawaii, Indonesia, France and Fiji. Regarding the origin of surfing, Nendel believes that surfing is an adventure activity for Hawaiians, an ancient sport, and has a profound impact on local life [4]. As for the formation of surfing destinations, Nardini studied the quality of the beaches that surfing relied on and proposed the basic conditions required for the development of surfing sports and how to maintain a suitable ecological environment [5]. Widener took Bali as an example to study the typical climatic and geographical features of surfing resorts and concluded that most of the surfing resorts are located in the coastal monsoon area, which is warm and humid and has frequent ocean currents. Benefiting from the long coastline bordering the Mediterranean Sea, the Atlantic Ocean and the English Channel, France has advantaged natural beaches. Among them, the southwest coast is widely sought after by surfers, and the famous wave spots include Crozon Morgat and Pampelonne. These excellent surfing spots provide superior natural basic conditions for surfing tourism [6]. Whitcomban took Hawaii as a case to study the relationship between indigenous culture and surfing culture and found that the native culture of Hawaii had a deep influence on the representation of surfing culture. For example, surfboard and swimsuit patterns used indigenous language pictures as logo designs. The government has also started to protect the healthy development and inheritance of indigenous culture and surfing culture. The combination of indigenous culture and surfing tourism has become an important feature of local tourism [7]. Fiji, a famous surfing destination, has a humid climate, high-quality soft sandy beaches, and well-developed tourism resources. Among them, the Mamanuka Islands consist of 20 small islands that provide diving and surfing activities. By taking Fiji as an example, Taylor concluded that the facilities required for tourism in surfing resorts include accommodation facilities, transportation facilities, recreational and sports facilities, and health care facilities. As a result, Tavarua Island is known as a typical demonstration site for the sustainable development of surfing tourism [8].

Hainan is the first place in China to launch surfing sports. The bay has superior natural conditions and excellent wave spots [9]. However, different from the rich practice abroad, there are still few studies on surfing tourism in mainland China, mainly focusing on countermeasures such as surfing tourism market development and surfing management. The corresponding theoretical research needs to be further strengthened. Empirical research on tourist experience has good practical significance for a deep understanding of surfing tourism.

### 2.2. The Embodiment of Tourism Behavior

Classical cognitive psychology believes that the acquisition of knowledge does not depend on the body, thus ignoring the mediating role of the body. With the development of psychological experiments, the important role of the body in knowledge acquisition has been gradually revealed, and the embodiment of knowledge acquisition, learning with the body, body awareness and the important role of the body in knowledge processing are emphasized [10]. The gaze theory in tourism experience emphasizes the important role of visual power in the experience. Urry’s tourism gaze theory [11] mostly interprets it as a visual experience while ignoring the role of gaze in coordination with other body senses. If we only understand the complexity of the tourism experience from the perspective of vision, it is bound to be one-sided, and we should pay more attention to the integrity of the body. The body is the mediator of emotional experience, and it was revealed in psychological experiments that there is a high correlation between physical behavior and emotional feelings [12].

Veijola pointed out the lack of physical issues in tourism research and further pointed out that tourists’ motivation to travel is to fully immerse the body in the current situation [13]. Cohen also emphasized that embodied emotion is an important theme of current tourism research [14]. Obrador highlighted the important role of touch in the quality of the tourism experience through the study of the tactile performance of tourists on the beach during holidays [15].

The development of embodied theory provides new possibilities for the exploration of the tourism experience [16,17,18]. Surfing tourism is a form of tourism experience that requires a high degree of physical participation. Under this theoretical impetus and realistic observation, this study conducted grounded theoretical research with surfing tourism text as the unit of analysis in an attempt to explore how the body is presented in the tourists’ surfing experience, what the dimensions of the embodied experience are and how it further affects the quality of tourists’ experience, in order to deepen the understanding of the surfing tourism experience theoretically.

## 3. Study Design

### 3.1. Study Questions and Methods

First of all, this study needed to answer the physical experience process and results of tourists’ surfing tourism behavior. For this question, we needed to go deep into the specific data mining and surfing context. Therefore, we adopted the bottom-up induction method to code, classify and abstract concepts so as to form an understanding of related issues. Secondly, there are relatively few studies on the surfing tourism experience from the perspective of embodied theory, while the most prominent feeling in the process of surfing tourism experience is body perception. It is necessary to put forward a theoretical model of surfing tourism embodied experience by taking root in practical problems. Finally, from the perspective of these two research questions, the grounded theory method is suitable for generative theory and has certain exploratory research, so it fully meets the problems to be solved in this research [19,20].

In order to answer the questions involved in this study, the researchers obtained textual data by collecting online travel notes and on-site interviews. In order to make up for the lack of on-site situation and feedback of immediate on-site experience in online travel notes, the researchers conducted random interviews at the same time and selected five representative interview texts as the source of data analysis. The interview followed the principle of volatilization, and the interviewees were given open answers under the condition of revealing their identities. The researchers did not guide the interviewees’ statements. The interview questions mainly involve basic information about the interviewees, why they choose surfing tourism, what expectations they have before surfing tourism, what feelings they have during surfing tourism, what effects surfing tourism has on their body feelings and other relevant open-ended answers. Based on the text data of online travel notes and on-site interviews, the researchers used the grounded theory method for technical analysis.

The objective of this study is to develop an embodied theoretical model of the surfing tourism experience. The grounded method was proposed by Glazer and Strauss in the 1960s and has been widely used in qualitative research in recent years. Its main purpose is to build a theory on the basis of empirical data [21]. This is a method of building theoretical models from the bottom up, that is, finding core categories reflecting the essence of phenomena on the basis of systematically collecting empirical data and then establishing connections between these categories and constructing relevant theories. Researchers, before the present research question, made no theoretical assumptions directly from the actual observation. This paper is based on the experience in material open coding and axial coding and chose the data type code, at the same time, in the process of constantly encoding compared with the original data; finally, raw data were conceptualized, and the theoretical propositions were processed. The grounded theory approach has a high degree of consistency with the thought of phenomenology, which emphasizes confronting the object itself on the philosophical level. In order to achieve the efficiency and accuracy of text data analysis, this study used the qualitative analysis software MAXQDA 2020 to conduct three-level coding and categorical analysis of text content.

### 3.2. Study Subjects and Data Collection

This study used the online travel notes published by surfing tourists and the interview materials with tourists as the analysis unit. The travel notes were selected according to the following criteria:

(1) The sample points come from a wide range of sources, and the surfing tourism cases involved are representative and cover different gender and age groups;
(2) The written records are detailed, with high authenticity and high credibility and dissemination power;
(3) The authors of travel notes are identified, texts written by tourists who accept sponsorships such as tourist destinations and travel websites are avoided, and objectivity is maintained.

First, the researchers searched and screened travel notes online and initially obtained 96 travel notes. According to the screening criteria, the two authors further identified 26 travel stories with rich content, mainly from China’s mainstream outdoor travel websites such as Qyer.com and Mafengwo 22 October 2021, as well as influential we-media platforms such as Sina blog. The interview materials were selected from 6 interview records of surfing tourists during the field survey in May 2022. There were a total of 32 texts in the two categories, including 17 female and 15 male, aged from the 60s to the 90s. After sample determination, the texts were individually numbered and input into MAXQDA 2020 analysis software, among which 28 were used for coding analysis and 4 (W4, W18, W25, F2) were used for theoretical saturation test. See Table 1 for sample characteristics.

## 4. The Study Process

### 4.1. Open Coding Procedure

The grounded theory method should go through the process of open coding, axial coding and selective coding. In the open coding stage, researchers read the text fully to ensure the familiarity of the data. Then, the meaningful codes were marked independently, and 760 basic concepts were determined after two comparisons and corrections. Then conceptualization was carried out, and a total of 21 categories were formed according to the semantic, logical relationship and concept connotation. It includes visual, aural, smell, taste, touch, body escape, body technique, body perception, emotional expression, body pain, emotional interaction field, body tools, other relationships, being close to nature, self-generation, behavioral metaphor, landscaping the other, hones the mind and body, enriched cognition, purifies the mind and self-constructs. An example of open coding is shown in Table 2.

### 4.2. Axial Coding Procedure

Formed by the axis type coding in open coding and based on the concept of axis type further compression coding concept relations, through repeatedly reading text data then the related concept, further compression were 21 categories into six main categories, which are, respectively, embodied perception, embodied awakening, embodied emotion, embodied extension, body representation and meaning construction. The relationship between each main category is shown in Table 3.

### 4.3. Selective Coding Procedure

**Figure 1:**
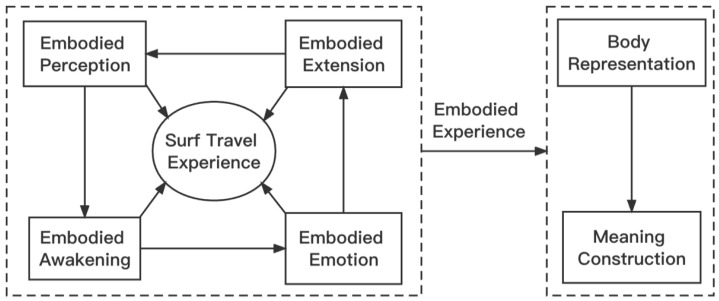
Typical relational structure of the main category.

The selective coding stage was based on the categories formed by open coding and axial coding, as shown in Figure 1, generating certain story lines and theoretical propositions to present the surfing experience process. After conceptualizing the relationship between the category concepts of surfing tourism experience, the core category was determined as embodied experience. The story line was developed around the core category of embodied experience, that is, tourists experience the process of embodied perception, embodied awakening, embodied emotion and embodied extension in the surfing tourism experience. Body sense is important in the surfing experience and location, and it uses the tourist’s physical representation as an intermediary to construct the tourist’s meaning. Modernity and one-dimensional life make the tourist’s body in a “dormant” state in the world of daily life. The requirements of surfing tourism for physical skills make the tourist’s physical experience fully awakened. After experiencing perception, awakening, emotion and extension, the body becomes an important carrier to represent the tourist experience and constantly constructs the meaning of self-identity and tourism experience under the interaction with oneself and others.

## 5. Model Building

**Figure 2:**
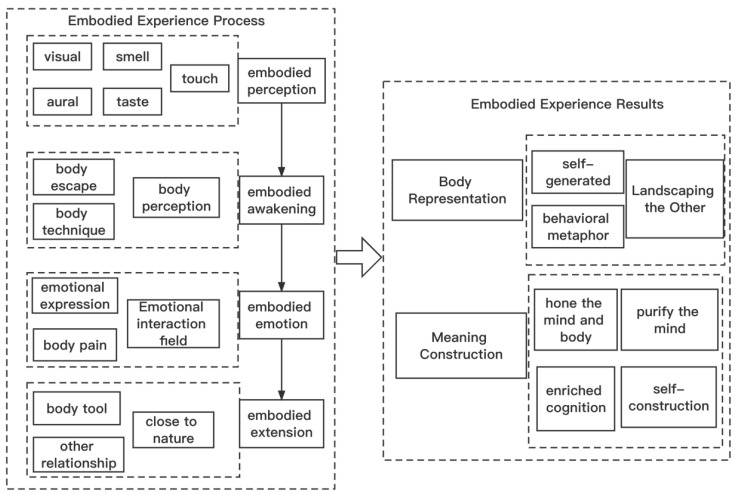
Embodied experience model of surfing tourism behavior.

After the saturation test of the retained text, no new category relationship was generated, indicating that the content of the data reached theoretical saturation. By centering on the core category and story line, this study constructed a model of the surfing tourism experience in Figure 2 to describe the embodied phenomenon of the surfing tourism experience and explain and interpret the process mechanism and social significance of its embodied experience.

### 5.1. Embodied Process of Surfing Tourism Experience

#### 5.1.1. Embodied Perception of Surfing Tourism Experience

Surfing tourism begins with the perception of the body and is comprehensively developed through the means of visual, aural, smell, taste and touch, providing the basis for the awakening of the body sense of tourists in the tourism world.

The visual experience is presented in the form of the tourist gaze. Wanning Riyue Bay has not only a good natural environment but also a rich community culture. Homestays and bars constitute the object of the visual gaze of tourists. “The signage design here is very distinctive, can feel the different surf culture” (W3). In addition to symbolic markers, the gaze of others is also a kind of understanding of surf culture for tourists. “The ronins here know they’re not like us. They have cool skin and cool hair” (F5).

The aural experience is an important component of the tourism experience [22,23]. The lapping sound of waves constructs the cognition of the ocean. Surfing in the water interweaves with the interactive sounds of others, condensing tourists’ experiences. “The waves in Riyuewan are very big, the sound is very powerful, the air is full of screams and cheers, in this environment, it is natural to want to try” (F1).

Smell experience is also presented in the surfing experience of Riyuewan, which constructs the local cognition of tourists to the surfing tourist destination. As the interview tourists mentioned, “When I come in, the air is full of the smell of hormones”; “The air here is very fresh, there is no general smell of the sea, I like it very much” (F4).

Taste experience and touch experience play an unforgettable memory in the surfing tourism experience. The salty taste of the sea is often felt when surfing. The reef at the bottom of the sea and the temperature of the sea make tourists realize the significance of the sea and waves.

#### 5.1.2. The Embodied Awakening of the Surfing Tourism Experience

Body escape is a kind of motivation for tourists to come to surfing tourism. The work pressure in modern society has increasingly become a one-dimensional life [24], and the boredom of traditional sightseeing and tourism methods contributes to tourists’ desire to awaken their physical senses. In the tourism experience, the surfing experience is a need to heighten the body and evade daily life. Surfing tourism “does not require much more special equipment, you only need a piece of plate, can rush to the sea, if you’re really immersed among them, will let you forget a lot of trouble” (Y11). When the tourists drop into the waves, the body’s presence and the situation unfolds.

Body technique is a special skill. Surfing tourism for beginners has a certain learning threshold; through the shore simulation of surfing action, some tourists experience the water skills. After imitating a series of movements, the surfing experience can be guaranteed. “Surfing is a bit adventurous and challenging, so I was willing to try it. The instructor showed us the basics and thought it was easy when we weren’t in the water. In fact, when in the water, it is difficult to board, and the pressure of the current also makes your body suffer a certain amount of pain, so we need to pay attention to their ability, otherwise there is still a certain risk” (w8).

Body perception when the tourist starts to surf the water through physical activities such as pushing the board, climbing the board, standing, etc., leaving “screaming bursts” and “feeling that the life force in the body is circulating endlessly” (F3). The body’s consciousness is affected by the individual and awakened by subjective efforts. At the same time, the changing waves cause troubles to the body, such as falling, injury, drowning, etc., causing the surfer to have physical reactions such as heavy breathing and sweating, which play a passive role in awakening personal body awareness.

#### 5.1.3. Embodied Emotion of Surf Tourism Experience

Emotional expression is the result of bodily reactions. In the surfing tourism experience, the worship of natural forces and the challenge of one’s own skills provide tourists with a way to express their emotions. From the analysis of the text data, it was found that tourists mostly use positive words to describe the tension and excitement during surfing. “I felt a wave coming from behind, and the whirring sound was quite shocking” (W9); “After I stood on the board for the first time, I screamed with excitement. It was indeed many attempts and failures. I was a little injured by being hit by a board, but it doesn’t matter. This experience is very rare. Although there are many people here, you can feel everyone’s passion. Overall, it is very exciting, fresh, and fun” (F1).

Body pain is a manifestation of the decline in the skill level of tourists in the surfing experience. In surfing, on the one hand, they have to face the unknown factors of the sea and waves. The changes in ocean currents make this sport full of adventure and danger. “There are many reefs in some places on the shore, which are very dangerous. I accidentally hit the reefs several times, and it hurts very much” (W5). Due to the special geographical environment and natural factors of Riyue Bay, the waves here are relatively large and have certain challenges. Ordinary tourists often experience surfing frustration and experience certain fatigue and suffering. “When I first started to go into the water, it was quite new, but this sport is really dangerous, and my physical strength is not enough, I felt very tired after playing for a while, but those who went into the water with me have to continue to play, I feel that it takes a long time to play. It would be a pity to have too much money to play. I’m actually quite tormented” (W9).

The emotional interaction field constructs the atmosphere of the surfing tourism experience. The body diagram of others provides a certain demonstration effect for surfers. In the process of living with waves, they are also getting along with people. In the process of the surfing experience, mutual influence and interaction form an important part of the water experience. “I actually just watch and learn from other people and they’re really willing to help me out. It’s hard to navigate a surfboard out in the ocean, but it really helps when people give you a few tips and practice a little bit and get the timing right. It’s actually nice for everyone to help each other like that” (F5).

#### 5.1.4. Embodied Extension of the Surfing Tourism Experience

Body tools are the extension of tourists’ physical skills. Surfing tourism mainly relies on equipment such as surfboards and swimsuits. Although the equipment seems simple, in fact, different materials and sizes of boards vary greatly in terms of surfing experience. For beginners, it is not suitable to use the smaller board; on the contrary, the larger board can give visitors a stable and safe surfing experience. More professional surfers are more willing to stretch their gear to the limit when looking for a place with the perfect waves than ordinary tourists. “You can see that these ronins are very particular about their clothes and the characteristics of their board. Their tools are different from ours, so I can immediately recognize those who play regularly. I took a lot of pictures with my camera, and when I came back to show them to others, they thought it was very special “(W8).

The relationship between others constitutes an important link in surfing tourism communication [25]. The surfing tourism community is an open and diversified area, which is full of fashion elements, and interpersonal communication is more simple and pure, without too many interest problems. While surfing, “Although I just met other tourists, we would exchange experience together. There was no coach next to me, and he would help me push the board, so that I could experience the fun of surfing” (F1). In the process of surfing tourism communication, the process from unfamiliar to familiar is relatively fast, and the cultural attribute here keeps narrowing the distance between people.

Close to nature is a return to human’s natural attributes. Most tourists live on land far away from the sea. In the process of surfing, they further deepen their understanding of the sea and waves. “Can be so unbridled, so close to nature, I can freely swim in the sea, Shouting, happy to sing a tune without tune, breathing the air with the smell of the sea, welcome the sunshine through the coconut leaves” (W6). This process of intimate contact with the Marine environment regenerates the awe of nature for tourists. “After leaving, I don’t miss surfing itself, but the sport provides a wonderful natural environment. I sigh and feel satisfied when I can blend in with nature” (W19).

### 5.2. Embodied Expression of Surfing Tourism Experience

Self-generation is a change brought to tourists by the surfing experience. Surfing enables tourists to re-understand the relationship between self and others, between self and nature, and between self and society (the relationship between self and society should include the relationship between self and others). On the one hand, the understanding of surfing culture allows tourists to broaden their cognition; on the other hand, the surfing experience allows tourists to reshape their outlook on life and re-examine various relationships. “Surfing is not just a culture. After learning about the sport, I found that there are a lot of positive worldviews, tolerance for life, and I’m not so obsessed with many things, but a more open-minded attitude towards life, which I think is what surf culture promotes. I agree with you “(W15).

The behavioral metaphor represents that surfing culture has a special “hippie” cultural meaning. More often, surfers will take the body in travel as the carrier of performance, display symbolic meaning through some daily behaviors, and build a cultural meaning space in the secular world. “I was curious why people who like surfing look and dress alike, so I thought they must share a common belief” (F3).

Landscaping the other points out that surf tourists will turn their gaze from the natural landscape to the others with them, as an expression of power desire, and gaze is a unique way of surfing tourists’ experience. “We just sit here in a daze, quietly watching those surfers, or standing on the folk balcony to admire travelers; watching those tourists wandering in the community looking for activities of their own interest” (W24).

### 5.3. The Meaning Construction of Surfing Tourism Experience

Honing the mind and body is a growing experience for tourists; after experiencing self-challenge to meet, tourists broaden their vision, pressure can be released and physical and mental quality can be exercised. Once tourists enter the water, they need to overcome multiple fears and unknowns, and constantly motivate themselves. After feeling the pleasure of surfing, they reap satisfaction. For professional surfers, being fully engaged in surfing is a “refreshing” experience, where the body and mind are deeply integrated with the current situation, resulting in great satisfaction. “I’m a surfer, and years of surfing have made me more able to face the difficulties in life and always think of the positive side of things, so I think I’m lucky to have learned the sport” (W25).

Enrich cognition is that every action in surfing tourism is its own physical skill, which helps tourists to fully and truly feel themselves and experience the greatness of nature. “Surfing can clear all the distracting thoughts from your body, including narrow-mindedness, pride, and only leave a strong curiosity. At this time, you are a child, full of the desire to know everything” (W5).

Purifying the mind is the process of surfing, which can compensate for the psychological deficiency brought by daily life so that the pain in the life world can be compensated, and in the process of observing the sea, people can reduce troubles, relax their nerves, and listen to their inner world, so as to gain a sense of tranquility and spiritual satisfaction. “I have a special awe for the sea, because the most expansive sea in the world is the sea, it can accommodate everything, and the soul can be purified here” (Y14).

Self-construction is the surfing tourists in the establishment of self-core value and self-realization and pleasant experience, constantly constructing the meaning of self and others, self and society. After surfing, participants perceived higher happiness, self-efficacy and competence because of the experience, feedback and sharing after surfing. “The coach said that people who can challenge success will have more confidence in themselves in the future, and those who fail in challenging challenges will not be afraid of failure in the future. It seems that at that time, I really understood this kind of truth. I feel that there is new hope in life, but it seems that there is only that. Feel like this for a few days” (F6).

## 6. Conclusions and Discussion

First, as the body theory researchers have stated, we used the body as the intermediary for the tourism experience [26]. In essence, surfing tourism behavior is a kind of embodied body experience. Body perception and awakening play an important role in the quality of the surfing tourism experience. Embodied imitation cognition theory points out that the body obtains cognition in practice [27], and the body experience plays an important role in the surfing tourism experience. Tourists deeply integrate with the surfing situation, perceive, understand and use the body with the participation of the body, and construct the relationship between themselves and others and society with the body experience.

Secondly, this study is not limited to the static perspective of surfing tourism behavior but the physical experience of surfing tourism into a dynamic development perspective to analyze. Tourism experience is characterized by mobility [28,29]. However, most studies on the mobility of tourism experience only start from the results and ignore the dynamics and development [30]. The fluidity of the surfing tourism experience is a dynamic process: it has experienced embodied perception, embodied awakening, embodied emotion and embodied extension. Each category has a certain interdependence. With the embodied perception of the surfing experience, tourists can perceive the external world with the help of multi-senses, such as vision, hearing, smell, taste and touch, to provide the basis for the awakening of body senses. Based on the needs of tourists’ bodies to escape from the daily living environment, embodied awakening uses body perception and body technology to obtain the pleasant experience of surfing tourism. With the awakening of tourists’ body perception, embodied emotion is presented. Through the process of expressing positive emotions and overcoming physical pain, tourists form an emotional interaction field. In this context, tourists further obtain a peak experience, thus improving the quality of the surfing tourism experience. Embodied experience needs to be assisted by the extension of the body; for example, through the realization of body instrumentalization, it is convenient for surfing tourists to better ride the waves, and through communication methods such as voice and interaction, the relationship with others is reconstructed. Under the closeness and awe of nature, the limitations of the body are understood. In this process, tourists fully mobilize their bodies, overcome physical obstacles, and obtain a pleasant tourism experience with the help of the awakening of body senses.

Finally, this study proposed the embodied experience model of surfing tourism behavior, expanded the scope of embodied theory in tourism experience, and proposed a new conceptual category and model. For example, surfing tourism needs the body to control tools to obtain experience, and the technical extension of the body is constantly stimulated and improved in the experience. Therefore, the embodied extension proposed further enriches the embodied theory of the tourism experience. In addition, the tourism experience is both representational and non-representational [31,32]. From the perspective of physical representation, the surfing experience generates a series of cultural identities. Tourists begin to gather in communities around the bay because of surfing culture, and they form symbolic symbols, which metaphorize their own value through a series of physical behaviors. The gaze of the surfing tourism experience to others constitutes a new landscape form of surfing land, and the image of a “Surfer” with cultural representation gradually constitutes a local landscape presentation. On the other hand, from the perspective of non-representative meaning construction, the surfing tourism experience has honed tourists’ physical and mental will, enriched their cognition, purified their minds in the process of getting along with nature, and gained self-value at the social level and the meaning of self-identification.

In addition to the contribution to the theory and knowledge, in facing China’s fast-growing surf tourism practical perspective, this study has certain guiding significance for the practice; the perception of tourists’ body experience, wake up and emotional shape can be targeted management and incentive measures for surf tourism product design and development to provide necessary theoretical support.

## Figures and Tables

**Table 1:** Basic sample information.

Respondent Number	Gender	Age	Source	Number of Words	Respondent Number	Gender	Age	Source	Number of Words
W1	female	18–30 years	Mafengwo 22 October 2021	1504	W17	female	18–30 years	Qyer.com 22 October 2021	4085
W2	female	18–30 years	Qyer.com 22 October 2021	567	W18	male	31–40 years	Qyer.com 22 October 2021	3754
W3	female	31–40 years	Mafengwo 22 October 2021	1293	W19	female	31–40 years	Sina blog	1870
W4	male	18–30 years	Qyer.com 22 October 2021	2544	W20	male	18–30 years	Mafengwo 22 October 2021	2975
W5	male	18–30 years	Qyer.com 22 October 2021	4584	W21	female	31–40 years	Sina blog	1086
W6	female	41–50 years	Mafengwo, 22 October 2021	1101	W22	male	41–50 years	Mafengwo 22 October 2021	3975
W7	male	18–30 years	Qyer.com 22 October 2021	2343	W23	female	31–40 years	Qyer.com 22 October 2021	2876
W8	female	18–30 years	Sina blog	3434	W24	male	31–40 years	Sina blog	3876
W9	male	51–60 years	Mafengwo 22 October 2021	755	W25	female	31–40 years	Mafengwo 22 October 2021	3304
W10	female	31–40 years	Sina blog	2383	W26	male	18–30 years	Sina blog	1954
W11	female	31–40 years	Qyer.com 22 October 2021	960	F1	female	18–30 years	On-site interview	4048
W12	male	18–30 years	Mafengwo 22 October 2021	2097	F2	female	51–60 years	On-site interview	7938
W13	male	31–40 years	Qyer.com 22 October 2021	1086	F3	male	41–50 years	On-site interview	3007
W14	female	18–30 years	Mafengwo 22 October 2021	2076	F4	female	31–40 years	On-site interview	4566
W15	female	18–30 years	Qyer.com 22 October 2021	3098	F5	male	18–30 years	On-site interview	2349
W16	male	18–30 years	Sina blog	1029	F6	male	18–30 years	On-site interview	1980

**Table 2:** Examples of open coding procedure.

Respondent Number	Original Statement	Conceptualization	Categorization
W5	I am really busy with work and lack exercise. I feel really burnt out. This time I came to Hainan to experience surfing. I think it is very good.	body escape	embodied awakening
W21	I am more adventurous and willing to try new things.	body technique	
W14	Before I came here, I watched a lot of guides, and I followed the video to do some basic moves at home. This time I also found a coach. Although it was really difficult to get on the board at first, I played for two days and finally succeeded.		
F4	After surfing, I found out that I still have this talent, and I found a sport again.	body perception	
W2	When I was in the water, although it was very uncomfortable to choke on the water, the good thing was that I could swim. I couldn’t surf, so I thought I was swimming.		

**Table 3:** Main categories formed by axial coding.

Main Category	Corresponding Category	Representative Code
embodied perception	visual aural smell taste touch	blue ocean the sound of waves lapping taste of the sea salty sea water hard rock
embodied awakening	body escape body technique body perception	social stress, relaxation, leaving routine, boring life body fitness (body strength, endurance, coordination), surfing skills (boarding, standing, catching waves), pre-charge training, surfing equipment active wake-up (boarding/catching waves/body awareness), passive wake-up (choking water)
embodied emotion	emotional expression body pain emotional interaction field	spectacular, wild, amazing, diverse, dangerous tired, difficult, tormented, painful, exhausted positive emotions, negative emotions
embodied extension	body tools other relationship close to nature	surfboard, camera communicating with surfers, helping people, staring at others get close to nature, feel the sea, play in the water
body representation	self-generation behavioral metaphor landscaping the other	discovery, growth, challenge, embodied, becoming try, meaning, incredible staring ronin, body aesthetics
meaning construction	hone the mind and body enriched cognition purify the mind self-construction	exercise, hone will, self-challenge, self-motivation embodied cognition, seeing the real world, novelty, freshness, vision purify the mind, heal the mind, relax the mind and body, soothing seek yourself
